# Is Iron Supplementation Associated with Infant Mortality in Sub-Saharan Africa and Does Birth Weight Modify These Associations?

**DOI:** 10.3390/nu17101696

**Published:** 2025-05-16

**Authors:** Yibeltal Bekele, Bircan Erbas, Mehak Batra

**Affiliations:** 1 School of Psychology and Public Health, La Trobe University, Melbourne, VIC 3086, Australia; y.bekele@latrobe.edu.au (Y.B.); b.erbas@latrobe.edu.au (B.E.); 2 School of Public Health, Bahir Dar University, Bahir Dar 79, Ethiopia

**Keywords:** iron supplementation, neonatal death, post-neonatal death, sub-Saharan Africa

## Abstract

**Background:** Iron supplementation during pregnancy is associated with several health benefits, including a reduced risk of maternal anaemia and improved neonatal outcomes such as lower rates of low birth weight, infection, and anaemia in infancy. However, its impact on neonatal and post-neonatal mortality remains unclear in resource-limited settings, where adherence to maternal iron supplementation is low. This study examined the association between maternal iron supplementation and neonatal and post-neonatal mortality and explored whether low birth weight (LBW) modifies those associations. **Methods:** This cross-sectional study utilised Demographic and Health Survey data collected between 2015 and 2023 from 26 sub-Saharan countries, including 287,642 neonates and 279,819 post-neonates. The primary outcomes were neonatal deaths (within 28 days) and post-neonatal deaths (between 29 days and 12 months). These outcomes and the exposure variables of iron supplementation and its duration were based on maternal recall. Adjusted odds ratios (aORs) with 95% confidence intervals (CIs) were estimated using generalised linear mixed models, with stratification by LBW. **Results:** There was no significant association between maternal iron supplementation and neonatal mortality (aOR = 1.07; 95% CI: 0.86, 1.34). However, the interaction between LBW and iron supplementation was statistically significant (*p* = 0.04). Among the LBW infants, the absence of iron supplementation increased the odds of neonatal mortality by 68% (aOR = 1.68; 95% CI: 1.14, 2.47), while supplementation for ≥90 days reduced the odds by 45% (aOR = 0.55; 95% CI: 0.35, 0.84). For post-neonatal mortality, lack of iron supplementation increased the odds by 25% (aOR = 1.25; 95% CI: 1.01, 1.56), whereas supplementation for ≥90 days reduced the odds by 27% (aOR = 0.73; 95% CI: 0.57, 0.93). **Conclusions:** Maternal iron supplementation was associated with lower post-neonatal mortality and improved neonatal survival among LBW infants. These findings suggest that iron intake may support infant survival, particularly in vulnerable populations.

## 1. Introduction

Globally, approximately 2.5 and 1.6 million babies die annually during the neonatal (within 28 days) and post-neonatal (between 29 days and 1 year) periods [1]. Low-income countries (LICs) account for 25% of neonatal mortality and 29% of under-five deaths globally [2]. Despite global reductions in neonatal mortality since 1990, progress in sub-Saharan Africa has lagged, achieving only a 39% decrease in neonatal deaths compared to 52% overall [2]. Projections indicate that 27.8 million neonatal deaths will occur between 2018 and 2030. During this period, the proportion of neonatal mortality within the overall under-five mortality is expected to rise from 47% in 2017 to 53% by 2030 [3]. Significant political and donor efforts under the Millennium Development Goals (MDGs) and Sustainable Development Goals (SDGs) have aimed to reduce neonatal and under-five mortality [4]. However, neonatal mortality in sub-Saharan Africa remains a critical challenge, with rates of 27.2 per 1000 live births, over seven times higher than in North America (3.6 per 1000 live births) [2]. While some regions are on track to meet the SDG 3 targets by 2030, most sub-Saharan countries are unlikely to achieve these goals, necessitating focused intervention in this region [5].

Neonatal and post-neonatal mortality rates reflect a country’s social welfare, governance, and economic status, with low-income nations facing the highest rates due to challenges in terms of system barriers and limited resources [6,7,8,9]. These deaths result from a combination of perinatal, environmental, and socio-demographic factors, including inadequate nutrition, infections, birth complications, and poverty [10,11,12,13]. Current evidence suggests that intrauterine factors significantly contribute to neonatal death, while environmental factors play a larger role in post-neonatal mortality [14,15]. Addressing maternal nutrition is critical, as deficiencies like iron deficiency remain widespread, especially in LICs [16,17]. Iron is essential for growth, immune function, and early development, and its deficiency exacerbates vulnerability to congenital malformations and infections, which are among the leading causes of neonatal and post-neonatal mortality [18]. Although the relationship between iron deficiency and neonatal and post-neonatal mortality is not yet firmly established, plausible biological mechanisms, such as impaired immune function and inadequate oxygenation, support this association [19]. To combat this, the World Health Organization and the United Nations Food and Agricultural Organization recommend food fortification and micronutrient supplementation, particularly iron [20]. In resource-limited settings with limited access to fortified food, oral iron supplementation during pregnancy is a key intervention to improve maternal and infant outcomes [21,22].

The relationship between iron supplementation and neonatal mortality has yielded inconsistent findings. A 2020 meta-analysis showed that prenatal supplementation containing 60 mg iron was associated with a reduced risk of neonatal mortality (RR = 0.80, 95% CI 0.68, 0.94) [23]. In contrast, another systematic review and meta-analysis of randomised control trials published in 2020, which included four studies in Europe and Asia, indicated that iron supplementation during pregnancy was not statistically associated with a reduced risk of neonatal mortality (RR = 0.91, 95% CI 0.71, 1.18). However, the quality of the evidence was deemed low due to several factors, including reporting bias, incomplete outcome data (due to attrition and loss to follow-up), detection bias, and high heterogeneity, all contributing to the imprecision of the findings. This review included data from China, Iran and Nepal, but no studies from Africa were included [24]. In comparison, our study improves upon these limitations by analysing a large, multi-country dataset from sub-Saharan Africa, adjusting for key confounders identified using a directed acyclic graph (DAG), stratifying by low birth weight (LBW), and employing generalised linear mixed models to account for clustering. These design features strengthen both the internal and external validity. Recent African studies examining the relationship between iron supplementation and neonatal mortality are limited and methodologically heterogeneous. For instance, one study reported no significant association but grouped neonatal mortality with stillbirths and perinatal deaths, limiting the interpretability [25]. Another study using cohort data suggested a potential protective effect, though the estimates lacked precision and context-specific confounder control [26]. In contrast, several studies from South and Eastern Asia have reported significant reductions in neonatal mortality associated with iron or multiple micronutrient supplementation [27,28]. These divergent findings may be attributed to differences in the baseline anaemia prevalence, intervention delivery, healthcare access, and nutritional status. Given that sub-Saharan Africa faces disproportionate burdens of maternal anaemia, limited antenatal care access, and food insecurity [29,30,31], extrapolating evidence from other regions may not be appropriate. Our study addresses this regional evidence gap by generating locally relevant data on iron supplementation and neonatal outcomes, thus providing an essential contribution to targeted maternal health interventions in African contexts.

Vulnerable populations, such as low-birth-weight (LBW) infants, face significantly higher risks of death during the neonatal period compared to the post-neonatal period [32]. LBW infants have lower iron stores and higher iron requirements than those of normal birth weight [33]. A study by Domellöf M. (2017) estimated that LBW newborns require 1–2 mg/kg/day of iron, which is substantially higher than the iron needs of normal-birth-weight newborns [33]. Accounting for these possible disparities through LBW stratification ensures that any assessment of these associations accurately captures the differential impacts of prenatal iron supplementation on neonatal and post-neonatal outcomes. To our knowledge, no prior studies have examined the association between iron supplementation, including its duration, and neonatal or post-neonatal mortality, stratified by the LBW of infants in sub-Saharan Africa.

## 2. Materials and Methods

This analysis utilised Demographic and Health Survey (DHS) data from 26 sub-Saharan African countries collected between 2015 and 2023. The country-specific survey years are detailed in Supplementary Table S1. Launched in 1984, the DHS programme operates in over 90 countries globally and collects data on infant and child mortality, nutrition, maternal and child health, and HIV indicators [34]. The surveys target women of reproductive age (15 to 49 years) and their younger children, with the data collection led by the Inner-City Fund (ICF) in collaboration with the national governments [35]. A multi-stage cluster sampling technique was used to select the participants. The analysis included 287,642 mother–neonate pairs and 279,819 mother–post-neonate pairs. Data were collected using standardised questionnaires translated into local languages to ensure consistency. Trained data collectors and supervisors fluent in the local languages conducted the surveys, while field supervisors and central office staff ensured quality control through regular supervision [36]. 

### 2.1. Outcomes of the Study

The study outcome included the following:

1. Neonatal death: Defined as the death of a newborn within the first 28 days of birth and categorised as yes/no [37].
2. Post-neonatal death: Defined as the death of a newborn between 29 days and the first year of life, categorised as yes/no [37].

Data on neonatal and post-neonatal mortality in the DHS programme are calculated from the full birth history questionnaire. This questionnaire captures detailed information on every child a woman has ever given birth to, including the date of birth, sex, survival status, age (if alive), and age at death (if deceased). All that information was obtained through maternal recall [38].

### 2.2. Exposures

The exposure variables were as follows:

1. Iron supplementation during pregnancy, regardless of gestational age: Categorised as yes/no, based on maternal recall.
2. Duration of supplementation: Categorised as no supplementation, less than 90 days, or at least 90 days, also based on maternal recall. To remind the participants, data collectors showed sample iron tablets commonly used in the community to those unsure about their supplementation.

### 2.3. Other Variables

A directed acyclic graph (DAG) was utilised to identify potential confounders in the analysis [39]. Key covariates included in the DAG were residence, maternal age, educational status (mother and partner), socioeconomic status, ANC utilisation, parity, media exposure, maternal occupation, access to care, marital status, and breastfeeding. Variables such as maternal BMI, gestational weight gain, pregnancy complications, genetics, and nutrient metabolism were considered but were not available in the dataset and are acknowledged as unmeasured confounders in the limitations. Based on the DAG and available data, the minimum sufficient adjustment set included maternal age, maternal education, maternal occupation, ANC use, residence, access to care, partner education, wealth index, and marital status (Supplementary Figure S1).

### 2.4. Data Analysis

The participant characteristics and the distribution of neonatal and post-neonatal deaths were described using the chi-square test, as all the variables were categorical. Due to the hierarchical nature of the data, which violated the independence assumptions of logistic regression, a generalised linear mixed model (GLMM) allowed the assessment of the association between iron supplementation and neonatal and post-neonatal death separately. To account for the non-independence of data within the clusters, a random effect was included in the model. The clustering variable (enumeration area: defined as the smallest geographic unit) was considered as a random effect, whereas variables such as the age of the mother, ANC service utilisation, residence, maternal educational status, partner educational status, family income, marital status, media exposure, maternal working status, and access to health care services were considered fixed effect variables.

A stratified analysis by low birth weight (LBW) was conducted based on a priori biological plausibility, given the increased physiological iron demands and reduced iron stores in LBW newborns compared to those of normal birth weight [33]. This approach aimed to provide deeper insight into how the associations between iron supplementation, its duration, and mortality outcomes may differ by birth weight, while minimising residual confounding [40]. The interaction terms between iron supplementation or its duration and birth weight (categorised as LBW or not) were included in the adjusted models. Interaction terms between iron supplementation (or its duration) and birth weight were included in the adjusted models. The adjusted odds ratio (aOR) and 95% CI were used to report the findings. Statistical analysis was conducted using STATA 18 [41], with a *p*-value ≤ 0.05 considered statistically significant.

## 3. Results

Table 1 and Table S2 summarise the participants’ descriptive statistics. The overall prevalence of neonatal death was 2.72% (*n* = 7818), while that of post-neonatal death was 1.83% (*n* = 5106). Neonatal death was higher among women who did not take iron supplementation (2.46%, *n* = 913) compared to those who did (1.81%, *n* = 2754, *p* < 0.001). Neonatal death’s prevalence varied significantly across the supplementation duration (*p* < 0.001), with the lowest prevalence observed among women taking supplements for ≥90 days (1.69%, *n* = 1117) compared to those without supplementation (2.46%, *n* = 913). A similar trend was seen for post-neonatal death (Table 1).

Age was a significant factor (*p* < 0.001), with a higher neonatal death prevalence in the extreme age groups, particularly among women aged 45 to 49 years (4.17%, *n* = 260) and 15 to 19 years (3.67%, *n* = 624). Socioeconomic factors played a critical role, with a lower neonatal and post-neonatal death prevalence seen among women with higher education levels (neonatal: 2.07%, *n* = 253; post-neonatal: 0.90%, *n* = 108) compared to those with no formal education (neonatal: 2.87%, *n* = 3164; post-neonatal: 2.22%, *n* = 2377). Similarly, increasing household wealth was significantly associated with a decline in the neonatal and post-neonatal death prevalence (*p* < 0.001) (Table 1).

Among LBW newborns, the neonatal death level was 4.46%, significantly higher than that of non-LBW newborns (1.29%, *n* = 1732; *p* < 0.001). Similarly, post-neonatal death was also higher among LBW newborns (2.32%, *n* = 346) compared to non-LBW newborns (1.18%, *n* = 1564; *p* < 0.001) (Table 1).

### 3.1. Neonatal Mortality

In a crude analysis, the absence of iron supplementation during pregnancy was associated with higher odds of neonatal mortality (cOR = 1.37; 95% CI: 1.24, 1.52; *p* < 0.001) compared to women who received supplementation. Taking supplementation for at least 90 days during pregnancy was associated with significantly lower odds of neonatal mortality compared to not taking it (cOR = 0.68; 95% CI: 0.60, 0.76; *p* < 0.001). In the adjusted analyses, the odds of mortality were 7% higher without supplementation (aOR = 1.07; 95% CI: 0.86, 1.34; *p* = 0.53), though this result was not statistically significant. Similarly, supplementation for ≥90 days did not show a significant reduction (aOR = 0.95; 95% CI: 0.74, 1.22; *p* = 0.71) (Table 2).

The interaction between LBW and iron supplementation (yes/no), or its duration (no/<90 days/≥90 days), was statistically significant (*p* = 0.04 and *p* = 0.02, respectively). The stratified analysis revealed that women who did not receive supplementation and had an LBW infant had 68% higher odds of neonatal mortality (aOR = 1.68; 95% CI: 1.14, 2.47; *p* = 0.01). In the same subgroup, supplementation for ≥90 days was significantly associated with a 45% reduction in the odds (aOR = 0.55; 95% CI: 0.35, 0.84; *p* = 0.01), while taking the supplement for <90 days reduced the odds by 38% (aOR = 0.62; 95% CI: 0.41, 0.94; *p* = 0.02) compared to those who did not take the supplement (Table 3).

### 3.2. Post-Neonatal Mortality

The absence of iron supplementation during pregnancy was significantly associated with higher odds of post-neonatal mortality in both the crude and adjusted analyses (cOR = 1.47; 95% CI: 1.32, 1.65; *p* < 0.001; aOR = 1.25; 95% CI: 1.01, 1.56; *p* = 0.04). In contrast, supplementation for ≥90 days was associated with lower odds of post-neonatal death (cOR = 0.58; 95% CI: 0.51, 0.66; *p* < 0.001; aOR = 0.73; 95% CI: 0.57, 0.93; *p* = 0.01) compared to no supplementation. Taking the supplement for less than 90 days was not significantly associated with reduced odds of post-neonatal death (Table 2).

The interaction between LBW and iron supplementation (yes/no), or its duration (no, <90 days or ≥90 days), on post-neonatal mortality was not statistically significant (*p* = 0.35 and *p* = 0.52, respectively). The stratified analysis showed no significant association between the absence of supplementation (aOR = 1.09; 95% CI: 0.65, 1.81; *p* = 0.75) or its duration (aOR = 0.78; 95% CI: 0.44, 1.38; *p* = 0.40) and post-neonatal mortality among LBW infants (Table 3).

## 4. Discussion

This study used a large, multi-country dataset to examine the association between maternal iron supplementation and neonatal and post-neonatal mortality in sub-Saharan Africa. Protective effects were observed among mothers who took iron supplements at any point during pregnancy, though stronger effects were seen with longer durations of use. Among LBW infants, the absence of maternal iron supplementation was associated with markedly elevated odds of mortality, with the highest odds observed during the neonatal period (68%) and notable odds also identified during the post-neonatal period (9%). While this study primarily focused on sub-Saharan Africa, its findings have broader relevance for other resource-constrained settings worldwide. By investigating the impacts of prenatal iron supplementation in regions with limited access to healthcare and nutrition, this research aims to provide insights that inform targeted interventions to reduce neonatal and post-neonatal mortality in underserved regions.

### 4.1. Neonatal Mortality

Maternal iron supplementation was associated with reduced odds of neonatal mortality, as expected, but the association was not statistically significant, including those supplemented for at least 90 days. This contrasts with a previous meta-analysis, which included five studies and found that prenatal supplementation with 60 mg of iron was associated with a 20% reduction in neonatal mortality [23]. In our study, the lack of statistical significance may be partly due to the inclusion of ANC in the model, which may have attenuated the independent effect of iron supplementation. ANC is a key platform for delivering multiple maternal health interventions, including iron supplementation [42]. In our sample, 87.62% of ANC attendees received iron supplements. These findings highlight the importance of considering how integrated service delivery, such as through ANC, may confound or mediate the observed associations between specific interventions and health outcomes.

Among LBW infants, our results indicated that maternal iron supplementation during pregnancy, particularly when taken for longer durations, was associated with reduced odds of neonatal mortality. The observed discrepancy between the overall non-significant association and the significant protective effect of iron supplementation in the low-birth-weight (LBW) subgroup suggests potential effect modification by birth weight. This heterogeneity may be explained by physiological differences in iron metabolism between LBW and normal-weight infants. LBW newborns have limited iron stores at birth and higher postnatal iron requirements relative to their size, making them more susceptible to iron deficiency and its consequences [33]. In contrast, normal-weight infants tend to have more stable iron reserves and may not derive the same survival benefit from additional supplementation [43]. Mechanistic studies indicate that iron deficiency impairs oxygen transport and immune responses, particularly T-cell proliferation and cytokine production, which increases susceptibility to infections such as neonatal sepsis, a leading cause of neonatal mortality [44,45,46]. Iron supplementation in LBW infants may therefore provide critical immunological and oxygenation support during the early postnatal period, which could explain the observed subgroup effect. These findings underscore the essential role of iron supplementation in resource-limited settings such as sub-Saharan Africa, where the prevalence of LBW is high. Additionally, they emphasise the importance of stratified analyses and suggest the need for tailored public health strategies to optimise supplementation protocols for high-risk newborn populations.

### 4.2. Post-Neonatal Mortality

Among all the women, iron supplementation was significantly associated with reduced odds of post-neonatal mortality, especially when taken for at least 90 days. While evidence of this relationship remains limited, these findings reinforce the enduring significance of iron supplementation programmes in improving post-neonatal health outcomes, which contribute substantially to under-five mortality. Environmental and nutritional factors, such as poverty, malnutrition, and inadequate healthcare access, are recognised as the primary drivers of post-neonatal mortality [47,48]. Additionally, childhood illnesses such as pneumonia, diarrhoea, and measles, often linked to nutritional deficiencies and immature immune function, are leading causes of post-neonatal and infant mortality [49,50].

In this context, maternal iron supplementation plays a multifaceted role in improving survival outcomes. Beyond addressing maternal anaemia, it enhances foetal oxygenation, immune system maturation, and overall resilience to infections, thereby reducing vulnerability to post-neonatal illnesses [51]. Moreover, iron deficiency during pregnancy can induce maternal and foetal stress through pathways such as hypoxia and elevated norepinephrine levels, which increase the risk of preterm birth, a key determinant of post-neonatal mortality [37,52]. While iron supplementation may help mitigate these risks, the precise mechanisms underlying its protective effects remain an area for further research.

Interestingly, no significant association was found between iron supplementation and post-neonatal mortality among LBW newborns. This could suggest that extrauterine factors, including infections, inadequate postnatal nutrition, and poor healthcare access, may be stronger determinants of mortality in this subgroup [14]. Given that post-neonatal survival depends on a combination of nutritional, environmental, and healthcare factors, supplementation alone may not be sufficient to mitigate the risks for LBW infants. Evidence-based postnatal interventions such as complete childhood immunisation, exclusive breastfeeding during the first six months, and timely introduction of appropriate complementary feeding at six months have been shown to significantly reduce post-neonatal mortality [53,54,55].

While this study highlights a potential association between iron supplementation and lower neonatal and post-neonatal mortality, it is also important to acknowledge the potential risks associated with excessive iron intake. Emerging evidence suggests that high-dose iron supplementation may contribute to adverse maternal outcomes, including GDM, through mechanisms such as oxidative stress and systemic inflammation [56,57]. GDM itself has been associated with an increased risk of adverse birth outcomes, including neonatal mortality [58]. These findings suggest that excessive iron intake could offset some of the potential benefits. Therefore, public health recommendations should move beyond universal supplementation approaches and instead prioritise individualised strategies based on maternal iron status and risk profiles. Such an approach would help optimise outcomes while minimising potential harms.

### 4.3. Strengths and Limitations

The key strength of this study lies in its large sample size (*n* = 287,642 mother–neonate pairs and *n* = 279,819 mother–post-neonate pairs), which enhances the statistical power of the analysis and allows for more robust generalisation of the findings, particularly to low–middle-income countries. This study also benefits from a rigorous analytical approach that accounts for multiple potential confounders, strengthening the validity of the findings. However, there are several limitations to consider. First, the cross-sectional nature of this study precludes the establishment of temporal relationships, which limits our ability to draw causal inferences between iron supplementation, its duration, and neonatal or post-neonatal mortality. This design is also susceptible to reverse causality, as women with higher health awareness or better health outcomes may be more likely to report supplement use. Second, survival bias may be present, since only mother–infant pairs in which the mother was alive at the time of the survey were included. This may result in an underestimation of mortality rates. Third, reliance on maternal recall may introduce information bias, including both recall and misclassification bias, particularly concerning supplement intake and duration. Unmeasured confounders such as genetics, gestational weight gain, maternal BMI, infections, dietary patterns, and pregnancy-related complications may further influence the associations. To address these limitations, future research should adopt prospective cohort designs incorporating biomarkers and genotyping to capture biological variability. In addition, sensitivity analyses comparing self-reported supplement data with medical records could improve the accuracy and reliability of the findings.

### 4.4. Implications and Future Research Directions

These findings highlight the need for more granular analyses of iron supplementation, particularly in populations with high burdens of maternal anaemia and LBW births. The observed association may be subject to unmeasured confounding. An E-value of 1.34 (95% CI: 1.12 to 1.50) suggests that an unmeasured confounder associated with both the exposure and the outcome by a risk ratio of 1.34 could explain away the observed effect. This relatively low threshold indicates that the findings should be interpreted with caution and highlights the need for future studies, including randomised controlled trials, to better assess the causality. The pseudo R^2^ value of 0.59 indicates a moderate model fit but also points to remaining unexplained variation, further supporting the need to include additional relevant variables in future analyses. Additionally, future research could assess the dose–response relationships to determine whether higher or more sustained supplementation throughout pregnancy yields additional benefits. Incorporating longitudinal studies that track maternal iron status from pre-pregnancy through postpartum would provide valuable insights into the optimal timing of supplementation and its sustained effects on infant survival. Additionally, exploring co-supplementation with other essential micronutrients, such as zinc, vitamin A, and folate, could enhance maternal and neonatal health by addressing multiple nutritional deficiencies contributing to poor outcomes. Strengthening community-based supplementation programmes is also critical to improving adherence and accessibility, particularly in resource-limited settings where coverage gaps remain challenging. Ultimately, expanding comprehensive maternal and infant nutrition strategies will be essential to achieving the SDG targets for child mortality reduction, particularly in low-income regions where the burden remains disproportionately high.

## 5. Conclusions

This study highlights the association between prenatal iron supplementation and reduced odds of neonatal and post-neonatal mortality, especially among LBW infants. It also emphasises the complex factors influencing neonatal survival and supports the need for targeted maternal nutrition interventions. To optimise maternal and child health outcomes, it is crucial to address the gaps in supplementation adherence, integrate co-micronutrient strategies, and improve data collection. Continued research is essential to inform evidence-based policies to improve child survival in resource-limited settings such as sub-Saharan Africa.

## Figures and Tables

**Table 1:** Neonatal and post-neonatal mortality by participant characteristics in sub-Saharan Africa.

Variable		Neonatal Death (*n* = 287,642)		*p*-Value	Post-Neonatal Death (*n* = 279,819)		*p*-Value
		Yes (%)	No (%)		Yes (%)	No (%)	
Iron supplementation. *n* = 188,990	Yes	2760 (1.8)	149,120 (98.18)	<0.001 *	1730 (1.16)	147,390 (98.84)	<0.001 *
	No	914 (2.46)	36,196 (97.54)		603 (1.67)	35,593 (98.33)	
Duration of iron supplementation *n* = 183,381	No	914 (2.46)	36,196 (97.54)	<0.001 *	603 (1.67)	35,593 (98.33)	<0.001 *
	<90 days	1430 (1.90)	73,701 (98.10)		944 (1.26)	72,757 (98.72)	
	≥90 days	1120 (1.70)	64,913 (98.30)		637 (0.98)	64,276 (99.02)	
Age *n* = 287,642	15–19	624 (3.67)	16,371 (96.33)	<0.001 *	369 (2.25)	16,002 (97.75)	<0.001 *
	20–24	1776 (2.76)	62,594 (97.24)		1144 (1.83)	61,449 (98.17)	
	25–29	1775 (2.35)	73,910 (97.65)		1221 (1.65)	72,689 (98.35)	
	30–34	1515 (2.49)	59,265 (97.51)		917 (1.55)	58,348 (98.45)	
	35–39	1249 (2.87)	42,299 (97.13)		742 (1.75)	41,557 (98.25)	
	40–44	619 (3.09)	19,411 (96.91)		405 (2.09)	19,006 (97.91)	
	45–49	260 (4.17)	5974 (95.83)		182 (3.05)	5791 (96.95)	
Educational status *n* = 287,642	Illiterate	3164 (2.87)	107,247 (97.13)	<0.001 *	2377 (2.22)	104,870 (97.78)	<0.001 *
	Primary	2517 (2.73)	89,558 (97.27)		1506 (1.68)	88,050 (98.32)	
	Secondary	1884 (2.58)	71,045 (97.42)		989 (1.39)	70,054 (98.61)	
	Higher	253 (2.07)	11,973 (97.93)		108 (0.90)	11,865 (99.10)	
Wealth index *n* = 287,642	Poorest	2132 (2.83)	73,251 (97.17)	<0.001 *	1577 (2.15)	71,673 (97.85)	<0.001 *
	Poorer	1781 (2.83)	61,056 (97.17)		1304 (2.14)	59,752 (97.86)	
	Middle	1592 (2.77)	55,860 (97.23)		969 (1.73)	54,889 (98.27)	
	Richer	1328 (2.66)	48,634 (97.34)		678 (1.39)	47,955 (98.61)	
	Richest	985 (2.34)	41,023 (97.66)		452 (1.10)	40,570 (98.90)	
ANC *n* = 189,619	Yes	3102 (1.81)	168,056 (98.19)	<0.001 *	1962 (1.17)	166,094 (98.83)	<0.001 *
	No	592 (3.21)	17,869 (96.79)		386 (2.16)	17,483 (97.84)	
Low birth weight *n* = 149,346	Yes	695 (4.46)	14,891 (95.54)	<0.001 *	346 (2.32)	14,545 (97.68)	<0.001 *
	No	1732 (1.29)	132,028 (98.71)		1564 (1.18)	130,463 (98.82)	

Footnote: *p*-value derived from the chi-square test, ANC: antenatal care, * statistically significant at *p*-value ≤ 0.05.

**Table 2:** Impact of iron supplementation and its duration on neonatal and post-neonatal death in sub-Saharan Africa.

Neonatal Death					
Exposure		cOR (95% CI)	*p*-value	aOR (95% CI)	*p*-value
Iron supplementation (*n* = 188,990)	Yes	1		1	
	No	1.37 (1.24, 1.52)	<0.001 *	1.07 (0.86, 1.34)	0.53
Duration of iron supplementation (*n* = 188,990)	≥90 days	0.68 (0.60, 0.76)	<0.001 *	0.95 (0.74, 1.22)	0.71
	<90 days	0.76 (0.69, 0.85)	<0.001 *	0.92 (0.73, 1.15)	0.46
	No	1		1	
**Post-Neonatal Death**					
Exposure		cOR (95% CI)	*p*-value	aOR (95% CI)	*p*-value
Iron supplementation (*n* = 185,316)	Yes	1		1	0.04 *
	No	1.47 (1.32, 1.65)	<0.001 *	1.25 (1.01, 1.56)	
Duration of iron supplementation (*n* = 185,316)	≥90 days	0.58 (0.51, 0.66)	<0.001 *	0.73 (0.57, 0.93)	0.01 *
	<90 days	0.75 (0.66, 0.85)	<0.001 *	0.85 (0.67, 1.08)	0.19
	No	1		1	

Footnotes: cOR: crude odds ratio; CI: confidence interval; aOR: adjusted odds ratio, * statistically significant at *p*-value ≤ 0.05; ANC, maternal education, birth weight, marital status, access to care, maternal age, partner education, residence, maternal occupation, parity and wealth index were controlled in the adjusted model.

**Table 3:** Stratified analysis of iron supplementation and its duration on neonatal and post-neonatal death by birth weight.

**Neonatal death**						
		**LBW (birth weight < 2500 g)** (*n* = 8839)		**No LBW (birth weight ≥ 2500 g)** (*n* = 8554)		**Interaction *p*-value**
Exposure		aOR (95% CI)	*p*-value	aOR (95% CI)	*p*-value	
Iron supplementation	Yes	1	0.01 *	1	0.38	0.04
	No	1.68 (1.14, 2.47)		0.88 (0.68, 1.15)		
Duration of iron supplementation	≥90 days	0.55 (0.36, 0.87)	0.01 *	1.18 (0.88, 1.58)	0.27	0.02
	<90 days	0.62 (0.41, 0.94)	0.02 *	1.10 (0.84, 1.44)	0.50	0.09
	No	1		1		
**Post-neonatal death**						
		**LBW (birth weight < 2500 g)** (*n* = 85,381)		**No LBW (birth weight ≥ 2500 g)** (*n* = 84,631)		
Exposure		aOR (95% CI)	*p*-value	aOR (95% CI)	*p*-value	*p*-value
Iron supplementation	Yes	1	0.75	1	0.04	0.35
	No	1.09 (0.65, 1.81)		1.29 (1.02, 1.65)		
Duration of iron supplementation	≥90 days	0.78 (0.44, 1.38)	0.40	0.72 (0.55, 0.93)	0.01	0.52
	<90 days	1.04 (0.60, 1.78)	0.90	0.81 (0.62, 1.06)	0.13	0.26
	No	1		1		

Footnote: maternal education, access to care, marital status, maternal age, partner education, residence, parity, maternal occupation and wealth index were controlled; aOR: adjusted odds ratio, CI: confidence interval, * statistically significant at *p*-value ≤ 0.05.

## Data Availability

Data are available upon request from the DHS programme website at https://www.dhsprogram.com/data/available-datasets.cfm, accessed on 22 February 2024.

## Supplementary Materials

The following supporting information can be downloaded at: https://www.mdpi.com/article/10.3390/nu17101696/s1, Figure S1 presenting the directed acyclic graph (DAG) used for selecting controlling variables in this study. Table S1: List of included sub-Saharan African countries and survey years. Table S2. Neonatal and Post-neonatal Mortality by Participant Characteristics in Sub-Saharan Africa.
