# Supplementary material for: Is Iron Supplementation Associated with Infant Mortality in Sub-Saharan Africa and Does Birth Weight Modify These Associations?
Source: Nutrients. 2025 May 16;17(10):1696. doi: 10.3390/nu17101696 (PMC12114251; doi:10.3390/nu17101696)
Supplement: Supplementary file 1 [file nutrients-17-01696-s001.zip › nutrients-3602356-supplementary.pdf]

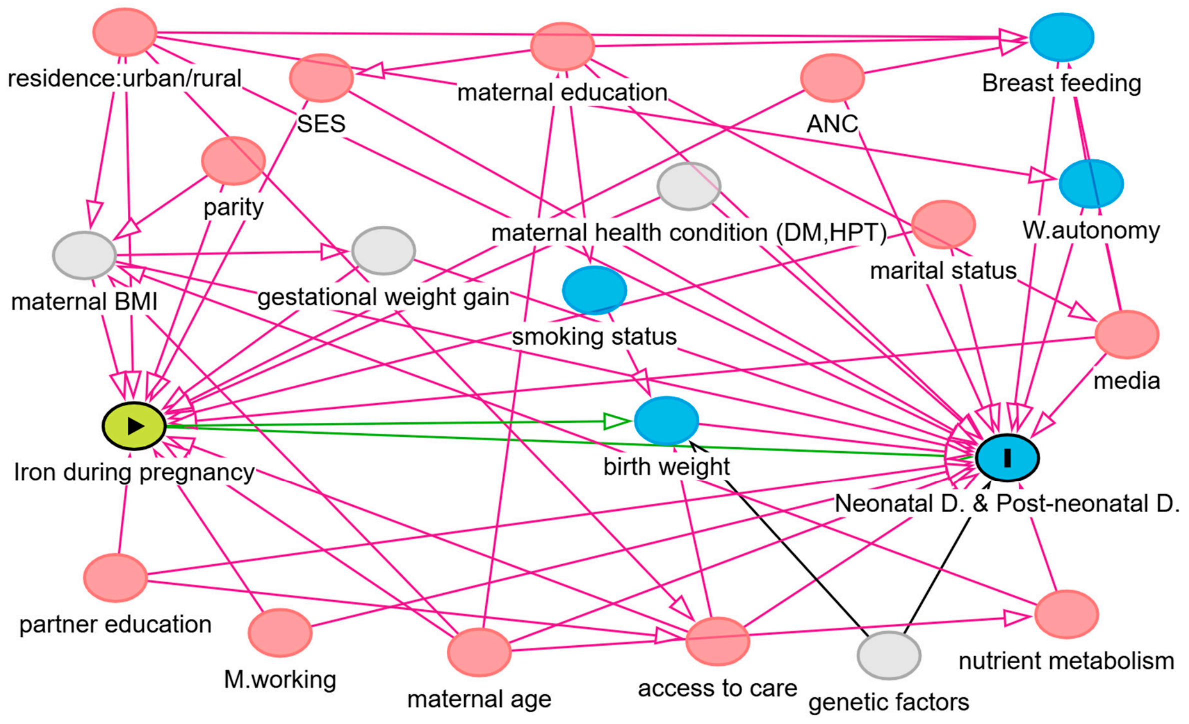

Supplementary Figure S1. Direct acyclic graph used to understand the causal pathways: ANC: antenatal care, SES: Socio-Economic Status, maternal BMI: Maternal Body Mass Index, M.working: Maternal Working Status, LBW: Low Birth Weight, DM: Diabetes Mellitus, HPT: Hypertension, w.autonomy: women autonomy Neonatal D: Neonatal Death and Post-neonatal D: Post-neonatal Death

Table S1: List of included sub-Saharan African countries and survey years	
**Countries**	**Survey Year**
Angola	2015/16
Benin	2017/18
Burkina Faso	2021
Burundi	2016/17
Cameroon	2018
Cote d'Ivoire	2021
Ethiopia	2016
Gabon	2019/20
Gambia	2019/20
Ghana	2022
Guinea	2018
Kenya	2022
Liberia	2019/20
Madagascar	2021
Malawi	2015/16
Mali	2018
Mauritania	2019-21
Nigeria	2018
Rwanda	2019/20
Senegal	2023
Sierra Leon	2019
South Africa	2016
Tanzania	2022
Uganda	2016
Zambia	2018
Zimbabwe	2015

Table S2. Neonatal and Post-neonatal Mortality by Participant Characteristics in Sub-Saharan Africa.

Variable		Neonatal Death (n=287, 642)		p-value	Post-neonatal Death (n=279,819)		p-value
		Yes (%)	No (%)		Yes (%)	No (%)	
Maternal working	No	2814 (2.60)	105,248(97.40)	0.004	1675 (1.59)	103,571(98.41)	<0.001
n=287,642	Yes	5004 (2.79)	174,576 (97.21)		3305 (1.89)	171,268 (98.11)	
Marital status	Single	528 (2.61)	19,699 (97.39)	0.031*	330 (1.68)	19,366 (98.32)	0.016
n=287,642	Married	5530 (2.75)	195,658 (97.25)		3465(1.77)	192,188 (98.23)	
	Living separate	1168 (2.53)	44,938 (97.47)		793 (1.76)	44,145 (98.24)	
	Widowed	109(2.98)	3554 (97.02)		88(2.48)	3466 (97.52)	
	Divorce	168 (3.10)	5252 (96.90)		91 (1.73)	5161 (98.27)	
	Separated	315 (2.85)	10,726 (97.15)		213 (1.99)	10,513 (98.01)	
Residence	Urban	2330 (2.54)	89,236 (97.46)	<0.001*	1222 (1.37)	88,014 (98.63)	<0.001*
n=287,642	Rural	5488 (2.80)	190,588 (97.20)		3758 (1.97)	186,830 (98.03)	
Accessing health care	Not a big problem	2873 (2.56)	109,482 (97.44)	<0.001*	1554 (1.42)	107,928 (98.58)	<0.001*
n=287,642	A big problem	4945 (2.82)	170,342(97.18)		3426 (2.01)	166,916(97.99)	
Partner Education	Illiterate	1717 (2.63)	63,477 (97.37)	<0.001*	986 (1.55)	62,488 (98.45)	<0.001*
n=247,105	Primary	448 (2.28)	19,174 (97.72)		198 (1.03)	19,175 (98.97)	
	Secondary	2760 (1.8)	149,120 (98.18)		1730 (1.16)	147,390 (98.84)	
	Higher	914 (2.46)	36,196 (97.54)		603 (1.67)	35,593 (98.33)	
Low birth weight	Yes	695(4.46)	14,891(95.54)		346 (2.32)	14,545 (97.68)	
N=149,346	No	1732 (1.29)	132,028 (98.71)	<0.001*	1,564 (1.18)	130,463 (98.82)	<0.001*
